# CT pulmonary angiography in patients with acute or chronic renal insufficiency: Evaluation of a low dose contrast material protocol

**DOI:** 10.1038/s41598-018-20254-y

**Published:** 2018-01-31

**Authors:** Mathias Meyer, Holger Haubenreisser, Christoph Schabel, Christianne Leidecker, Bernhard Schmidt, Stefan O. Schoenberg, Thomas Henzler

**Affiliations:** 1 Institute of Clinical Radiology and Nuclear Medicine, University Medical Center Mannheim, Medical Faculty Mannheim – Heidelberg University, Mannheim, Germany; 2 Department of Radiology, Duke University Medical Center, Durham, NC United States; 3 Imaging & Therapy Division, Siemens Healthcare, Forchheim, Germany

## Abstract

Adverse effects of intravenous contrast media (CM) in patients with renal risk factors and acute kidney injury are still controversially discussed. The aim of this study was to investigate whether dual-energy (DE) pulmonary CT angiography (CTPA) in combination with a noise optimized virtual monoenergetic imaging algorithm allows for a reduction of CM. This IRB-approved study comprised 150 patients with suspected pulmonary embolism (78 male; mean age 65 ± 17years). 50 patients with acute/chronic renal failure were examined on a 3^rd^ generation dual-source CT with an optimized DE CTPA protocol and a low CM injection protocol (5.4 g iodine). 100 further patients were either examined with a standard CTPA protocol or a standard DE CTPA (32 g iodine). For the DE CTPA virtual monoenergetic spectral datasets (40–100 keV) were reconstructed. Main pulmonary arteries at 50 keV and peripheral pulmonary arteries at 40 keV datasets provided the highest contrast-to-noise-ratio (CNR) for both the standard DE CTPA and the optimized protocol, with significantly higher CNR values for the standard DE CTPA protocol (p < 0.05). No pulmonary embolism was missed on the optimized CM protocol. DE CTPA utilizing image reconstruction at 40/50 keV allowed for a reduction of 84% in iodine load while maintaining CNR, which is especially important in patients with acute/chronic renal failure.

## Introduction

CT pulmonary angiography (CTPA), which requires the injection of iodinated contrast material (CM), is considered the gold-standard diagnostic modality in patients with suspected acute pulmonary embolism (PE)^1^. However, caution is warranted in patients with renal risk factors such as acute and chronic nephropathy. Guidelines of the European Society of Urogenital Radiology recommend to use the lowest amount of contrast medium necessary^2^. This may be subject to change since administration of intravenous CM in patients with renal risk factors is part of an ongoing controversial discussion and several recent studies have uniformly reported promising results regarding adverse renal reactions such as contrast induced nephropathy (CIN) or acute kidney injury (AKI). Aforementioned studies have indicated that CIN and AKI may be independent from the administration of intravenous CM among different risk groups^3–6^. Although those retrospective high quality studies may impact future guidelines, some limitations remain, including selection biases, limited information for patients undergoing several CT examinations within a few days and the impact of intravenous CM’s amount on acute kidney injury. Thus, if intravenous CM is essential for CT examinations, a reduction to a minimum amount of CM remains recommended, similar to the “*as low as reasonably achievable*” principle for radiation dose reduction^2^.

Over the last decade, low peak-kilovoltage (kVp) imaging between 70–80 kVp in combination with high-pitch acquisitions has allowed a significant reduction of CM for CTPA protocols with a total dose amount down to 9–21 g^7–9^. Low kVp imaging is currently limited to 70–80 kVp across all CT manufacturers due to a reduced efficiency of x-ray tubes with insufficient photon emission, increasing image noise at lower kVp levels. Dual-energy (DE) CTPA, which allows the calculation of virtual monoenergetic spectral (VMS) images at various kilo-electron volt (keV) levels with a minimum of 40 keV, can be used to promote iodine based contrast enhancement as an alternative CTPA imaging technique to further reduce CM. Studies have evaluated the feasibility of DE CTPA using a standard CM-, as well as a reduced CM-protocol^10–12^. However, these studies have used a first generation VMS software technique, which lacks noise compensation at low keV levels, thus limiting diagnostic image quality below 60 keV. Recently, a noise-optimized VMS algorithm was introduced to improve contrast-to-noise ratio at energy levels below 60 keV.

Therefore, the aim of this study was to investigate whether DE CTPA in combination with a recently introduced noise optimized VMS algorithm allows for a further reduction of CM in CTPA studies of patients with renal risk factors.

## Methods

### Study design

This prospective single-center study was approved by the institutional review board (Medical Ethics Committee II, Mannheim, Germany) and complies with both the Declaration of Helsinki and the Health Insurance Portability and Accountability Act (HIPAA). Informed consent was obtained from all participating patients.

In total, 150 patients were enrolled in this two-phase study. During the first part of the study 100 patients with suspected PE were equally (1:1 ratio) randomized either to a standard CTPA examination or a DE CTPA examination. Independently of a patient’s renal function a standard CM injection protocol was performed to evaluate non-inferiority of the DE CTPA protocol. The main objective of this first phase was to assess image quality (contrast-to-noise-ratio [CNR] and image noise) of VMS datasets between 40–70 keV compared to images of a standard non-DE CTPA protocol. Subjective and objective image quality were analyzed one month after the inclusion of the last patient. Subjective image quality was evaluated by two radiologists in a consensus reading (eight and four years of experience in chest CT) using a 5 point Likert scale as previously described^13^. Non-inferiority of the DE CTPA protocol was defined as a decrease in CNR of less than 5% of the pulmonary arteries and overall subjective image quality. In this first phase, the secondary objective was to evaluate diagnostic accuracy of VMS datasets between 40–70 keV of the standard DE CTPA in comparison to the 120 kVp virtual polyenergetic spectral (VPS) images. The diagnosis of PE and its severity were classified by two radiologists (T.H.; M.M.) according to recent guidelines^1,14^. Non-inferiority of the DE CTPA protocol was defined as *no false positive or negative PE finding on 40*–70 *keV VMS datasets*.

In a second phase, after non-inferiority of the DE CTPA protocol for VMS datasets between 40–70 keV was proven, 50 consecutive patients with renal risk factors underwent DE CTPA using the low CM-protocol. The main objective of this second study phase was to evaluate the objective image quality (CNR and image noise) of the low CM DE CTPA in comparison to the standard CTPA protocol. The secondary purpose of this second study phase was to evaluate the diagnostic accuracy of the low CM DE CTPA as a prerequisite to be implemented into clinical routine. The diagnosis of PE and its severity were classified as described above. One week after each scan, the same radiologists (T.H.; M.M.) analyzed the DE CTPA data in consensus regarding the presence or absence of PE (blinded to the previous rating). The standard of reference was established in consensus by both radiologists after each patient’s reading based on the clinical course, including response to therapy and diagnostic follow up, in compliance with the STARD criteria^15^. An exploratory objective of this study was to monitor serum creatinine levels before and 24 hours, 72 hours and 3 months after CTPA related CM application. Patients were questioned for re-administration of iodine CM during the follow-up period of 3 months. CIN was defined as an absolute increase in serum creatinine from baseline by ≥ 0.5 mg/dl, indicating acute impairment in renal function^16^.

### Examination Technique

The low CM DE CTPA protocol was performed on a 3^rd^ generation dual-source CT (DSCT) (SOMATOM Force, Siemens Healthineers, Forchheim, Germany) with the following scan parameters: tube A: 90 kVp tube voltage, no tin filter, 100 mAs reference tube current; tube B: 150 kVp tube voltage, 0.6 mm tin filter, 90 mAs reference tube current. Rotation time was 0.25 s, pitch 1.0 and the chosen detector collimation 192 × 0.6 mm for both tube systems. The contrast agent administered was iomeprol 400 mg Iodine/mL (mg I/mL, Imeron 400, Bracco Imaging S.p.A., Milan, Italy), diluted via an injector to a concentration of 30% to 70% 0.9% NaCl totaling 45 ml and 5.4 g iodine content. This was followed by a saline chaser of 30 ml. Flow rate was 3 ml/s through an antecubital vein.

The following scanning parameters for the standard CTPA protocol (Siemens SOMATOM Definition Flash, Healthineers, Forchheim, Germany) were used: 100 kV tube voltage, no tin filter, 140 mAs reference tube current, 0.28 s rotation time, 1.2 pitch, 128 × 0.6 mm detector collimation. The following scan parameters for the DE CTPA on the same scanner were used: tube A: 100 kV tube voltage, no tin filter, 90 mAs reference tube current; tube B: 140 kV tube voltage with tin filter, 80 mAs reference tube current. For both tube systems the rotation time was 0.28 s, pitch 0.55 and the chosen detector collimation 128 × 0.6 mm.

Vessel attenuation was achieved for both standard protocols by injecting 80 mL iomeprol 400 mg I/mL (32 g iodine content) followed by a saline chaser of 40 ml through an antecubital vein at a flow rate of 4 mL/s^17^. Both CTPA protocols utilized an automatic tube current modulation (CARE Dose4D, Healthineers, Forchheim, Germany).

The CT acquisition was timed by using bolus tracking for both CTPA protocols with a region-of-interest (ROI) placement in the pulmonary trunk. Once a threshold of 100 Hounsfield Units (HU) was reached (standard CTPA protocol triggering at 100 kVp and DE CTPA protocol triggering at 70 kVp), the scan automatically started after a 5 second delay, allowing for a breathing command (“gently breath in”). Table 1 summarizes scan acquisition parameters, contrast injection protocols and image reconstruction techniques for the three different CTPA protocols.

**Table 1:** Patient demographics, iodine load and dosimetric parameters.

	Standard CTPA protocol n = 50 [A]	Low CM DE CTPA protocol n = 50 [B]	Standard CM DE CTPA Protocol n = 50 [C]	p-value A vs B	p-value A vs C	p-value B vs C
Age [years]	64 ± 22	67 ± 10	67 ± 15	0.3013	0.3876	0.8412
Male	26	27	25	0.77	>0.99	0.8415
Body-mass-index (kg/m^2^)	26.1 ± 4.2	27.1 ± 3.1	27.9 ± 4.5	0.4232	0.3876	0.8764
Pulmonary embolism	7	7	6	—	>0.99	>0.99
Central pulmonary embolism	3	4	3	>0.99	>0.99	>0.99
Right ventricular dysfunction	2	2	2	>0.99	>0.99	>0.99
Normal creatinine clearance [>60 ml/min]	40	0	46	<0.0001	0.9796	<0.0001
Acute kidney failure	4	29	2	<0.0001	>0.99	<0.0001
Baseline creatinine level [mg/dl]	0.98 ± 0.2	1.37 ± 0.3	0.95 ± 0.4	<0.0001	0.3623	<0.0001
Creatinine level after 24 h [mg/dl]	1.03 ± 0.2	1.38 ± 0.4	0.99 ± 0.4	<0.0001	0.2221	<0.0001
Creatinine level after 72 h [mg/dl]	1.04 ± 0.2	1.35 ± 0.3	0.93 ± 0.5	<0.0001	0.0925	<0.0001
Creatinine levels after 3 Months [mg/dl]	1.01 ± 0.2	1.21 ± 0.3	0.93 ± 0.4	<0.0001	0.0667	<0.0001
Total amount of iodine [g]	32.0	5.4	32.0	<0.0001	—	<0.0001
CTDIvol [mGy]	4.65 ± 2.6	6.83 ± 3.4	7.91 ± 3.9	0.0003	<0.0001	0.0321
DLP [mGy*cm]	169.2 ± 91.4	243.3 ± 117.3	285.5 ± 123.1	0.0012	<0.0001	0.0215
Effective dose [mSv]	2.4 ± 1.3	3.4 ± 1.6	3.9 ± 1.7			

Note – CTPA = CT pulmonary angiography; DECT = dual energy CT; DLP = Dose length product; CTDI_vol_ = volume CT dose index.

### Image Reconstruction

**Figure 1:**
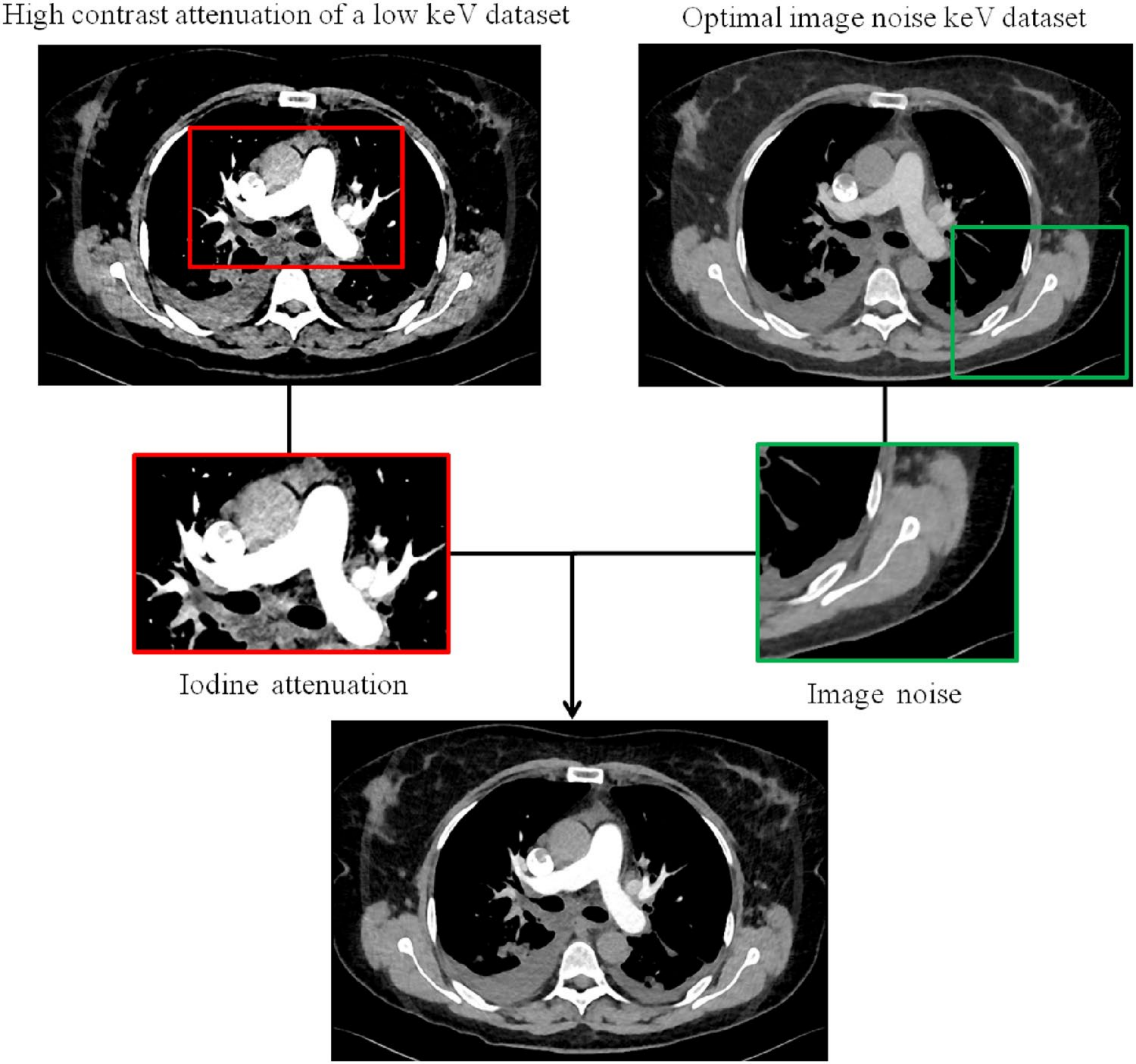
Simplified image reconstruction work flow of the monoenergetic reconstruction algorithm. A frequency-split technique is used which decomposes both the low keV images (in which iodine pixels have a high contrast to the surrounding tissue, typically at 40 keV) and images at higher keV (in which surrounding tissue has low image noise, typically at approximately 70 keV) into two sets of sub-images. In a next step, the lower spatial frequency stack at low keV is combined with the high spatial frequency stack at optimal keV from a noise perspective to combine the benefits of both images stacks.

For the DECT protocol 120 kVp VPS images were reconstructed at a weighing factor of 0.8 and 0.4 for the low CM and the standard DE CTPA protocol, respectively, and as recommended by the manufacturer. All DE images were reconstructed with a slice thickness of 1.5 mm in axial and coronal planes using a corresponding quantitative DE kernel (2^nd^ generation DSCT Q30f and 3^rd^ generation DSCT Qr40) and a soft tissue kernel for the standard CTPA protocol (I30f). All images were reconstructed using an iterative reconstruction (IR) algorithm. In detail, the 3^rd^ generation DSCT utilized a model based iterative reconstruction method (Advanced Model Based Iterative Reconstruction [ADMIRE] Siemens Healthineers, Forchheim, Germany) and the standard CTPA protocol a sinogram affirmed iterative reconstruction algorithm (Sinogram Affirmed Iterative Reconstruction [SAFIRE], Siemens Healthineers, Forchheim, Germany). The iterative reconstruction algorithm was set to a strength level of three for all three CTPA protocols, as recommended by the manufacturer and previously validated for low kV imaging of vascular structures^18^. The images were exported to a server based workstation (Syngo.via, Version VA30, Siemens Healthineers, Forchheim, Germany) for further post-processing and measurements in a timely manner. VMS images were calculated for DE datasets at energies between 40 and 100 keV with increments of 5 keV (Monoenergetic Plus, Siemens Healthineers, Forchheim, Germany). (Fig. 1)^19^.

### Objective Image Quality Assessment

Attenuation (HU) and image noise (standard deviation of attenuation) were measured for identical sized ROIs in three regions of the central pulmonary arteries (main, left and right pulmonary artery) and two peripheral pulmonary arteries (one lower and one upper lobe segmental artery) by a radiology resident (M.M), as previously described^13^. Further identical sized ROIs were placed in background air (for DE datasets in the VPS images) in order to assess background noise. These parameters were used to determine the SNR and CNR with the erector spinae muscle being the surrounding tissue^20^. For further analysis, the averages of the three central pulmonary arteries and the two peripheral pulmonary arteries were calculated.

### Subjective Image Quality Assessment

All technical and personal data were removed from the images prior to the subjective image quality assessment. Subjective image quality was analyzed by a board certified radiologist and a radiology resident (T.H., M.M.) both independently and in a consensus reading. This two-sided approach was chosen to evaluate inter-reader variability of the new contrast media protocol. Both radiologists rated the overall image quality (1 = poor, 2 = fair, 3 = moderate, 4 = good, 5 = excellent) and image noise (1 = unacceptable image noise, 2 = above average noise, 3 = average image noise, 4 = less than average noise, 5 = minimal image noise) on a 5 point Likert scale as previously described^13^ and in accordance with the criteria described for chest CT examinations in the European Guidelines on Quality Criteria for CT^21^.

### Radiation dose assessment

The volume CT dose index (CTDI_vol_) and dose length product (DLP) in the patient protocol were used to estimate radiation exposure. The conversion factor to effective organ dose was chosen according to the ‘European Guidelines for Multislice Computed Tomography (dose conversion coefficients of thoracic region: 0.0014 mSv Gy^−1^ cm^−1^;^22^).

### Statistical analysis

A two-sided binominal test with a significance level of 0.05 and a power of 0.8 were used for calculating the required sample size using the statistical software PASS 11, (LLC. Kaysville, Utah, USA. www.ncss.com).

Statistical analysis was performed using JMP 10.0 (SAS Institute Inc., Cary, NC, USA). Normally distributed data were identified using the Shapiro-Wilk W test. Continuous variables are presented as mean ± standard deviation and ordinal variables as median with a 25% to 75% interquartile range. Comparisons between CTPA groups and serum creatinine time points were either using either a two-way analysis-of-variance (ANOVA), if data were normally distributed, or a Kruskal-Wallis two-way analysis-of-variance, if data were not normally distributed. Subsequent Bonferroni correction was performed in order to account for multiple testing influences and Tamhane’s-T2 post-hoc testing, respectively, when variances were not equal in Levene’s statistics. For intra-patient comparison between VMS datasets repeated ANOVA measures using Dunnett’s test were performed. The consensus reading was used for statistical significance assessment between the three DSCT protocols. Sensitivity, specificity and accuracy were calculated on a per-patient base for the 40 keV and 50 keV VMS datasets of both DE CTPA protocols, as well as for the standard CTPA protocol. P-values < 0.05 were considered statistically significant throughout the entire study. A cohen’s kappa statistic was performed in order to assess inter-reader variability of subjective image quality. For all measurements indicating a significant difference a power analysis was performed, indicating a power >0.64.

## Results

### Patient demographics and dosimetric parameters

All scans have shown sufficient image quality and were rated as diagnostic. A total of 20 patients showed findings of PE, seven patients in the standard CTPA protocol group, six in the standard DE CTPA protocol group and seven in the low CM DE CTPA protocol group. In each of the CTPA groups, the PE was rated as severe in two patients (both 4%), as these patients showed right ventricular dysfunction on echocardiography. There was no significant difference in sex, age and body-mass-index between all three groups (all p > 0.05; compare Table 1). As expected by the study assignment, the number of patients with renal impairment (Modification of Diet in Renal Disease (MDRD) estimated glomerular filtration rate (GFR) >60 ml/min) was lower for both standard CTPA groups compared to the CM DE CTPA group (n = 40/46 and 0, respectively). The radiation dose was significantly higher for both DE CTPA protocols compared to the standard CTPA protocol (p < 0.05). Patient demographics, serum creatinine levels and radiation dose values are summarized in Table 1.

### Comparison of objective image quality of the standard CM DE CTPA images, the low CM DE CTPA images and standard CTPA images

**Figure 2:**
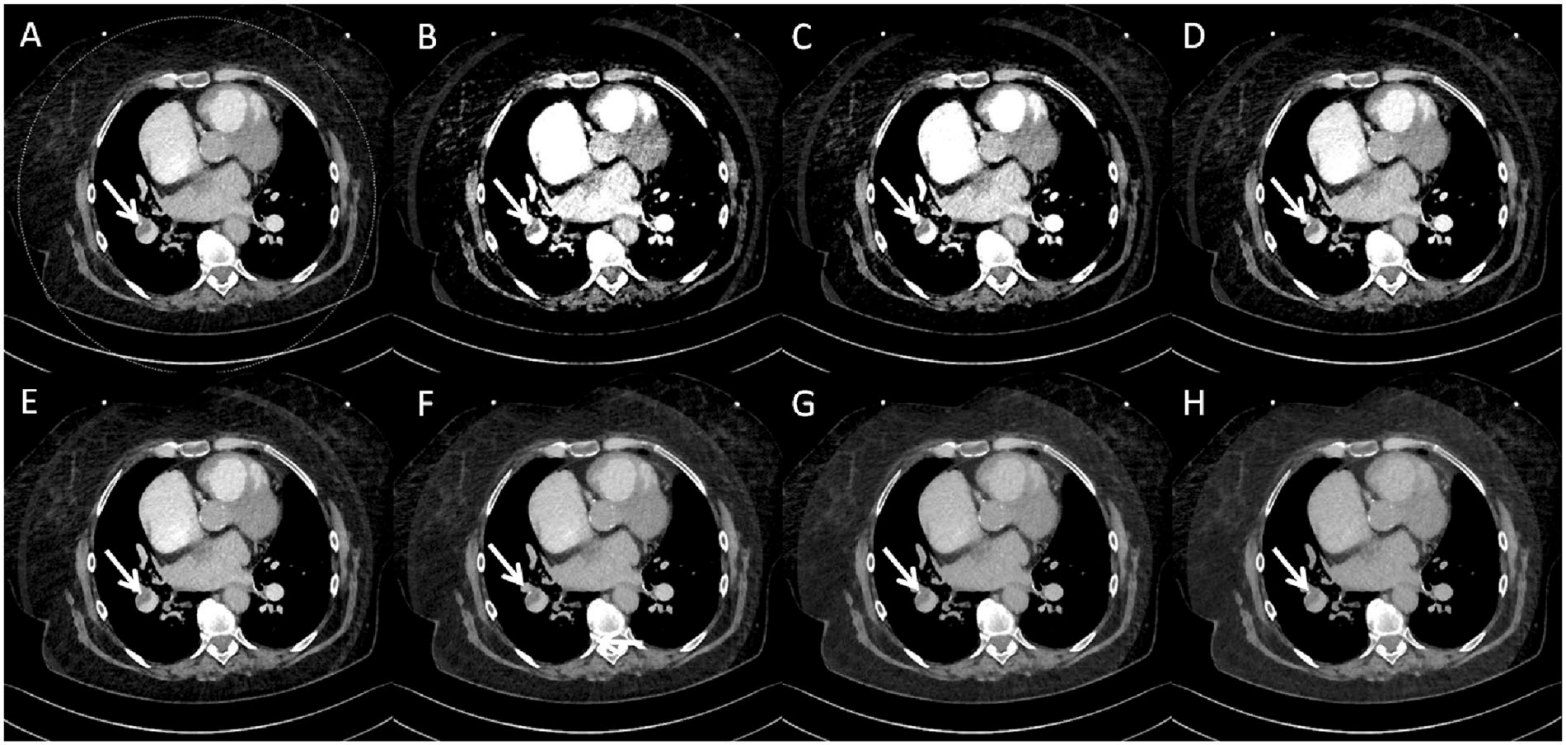
73-year-old woman with a peripheral pulmonary embolism (white arrows). Axial slices of main pulmonary arteries of a low contrast media dual-energy CTPA: (**A**) mixed 0.8-weighted virtual polyenergetic spectral (VPS) image, and virtual monoenergetic spectral (VMS) images at a level of 40 keV (**B**), 50 keV (**C**), 60 keV (**D**), 70 keV (**E**), 80 keV (**F**), 90 keV (**G**) and 100 keV (**H**).

**Figure 3:**
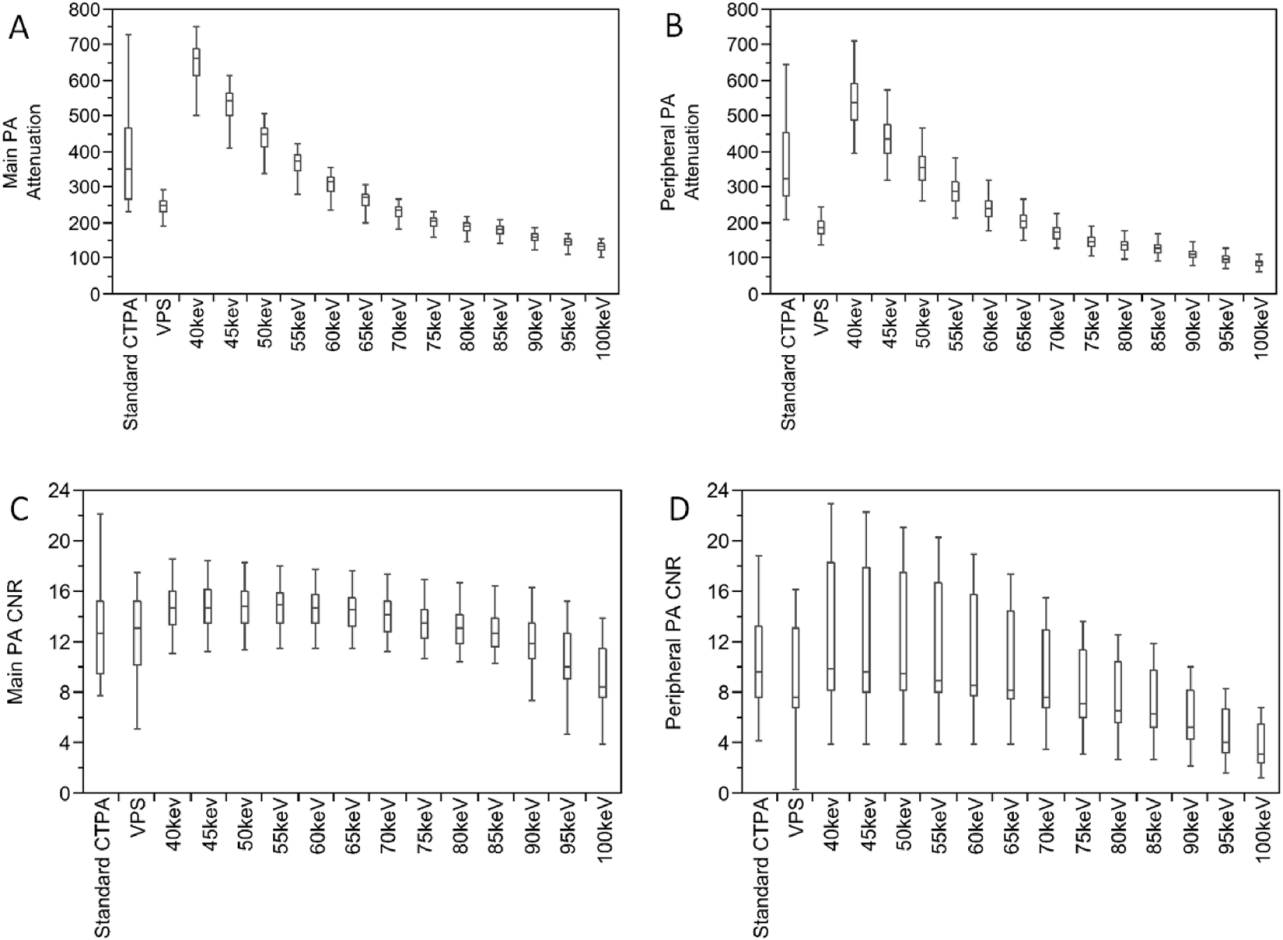
Box- and Whisker-Plots with values for attenuation (**A**,**B**) and contrast-to-noise-ratio (CNR; **C**,**D**) in the main pulmonary arteries (PA, **A**,**C**) and the peripheral PA (**B**,**D**) of a standard CTPA protocol, virtual polyenergetic spectral datasets (VPS) and virtual monochromatic spectral (VMS) datasets of a low contrast media dual-energy CTPA at nine-teen different energy levels.

**Figure 4:**
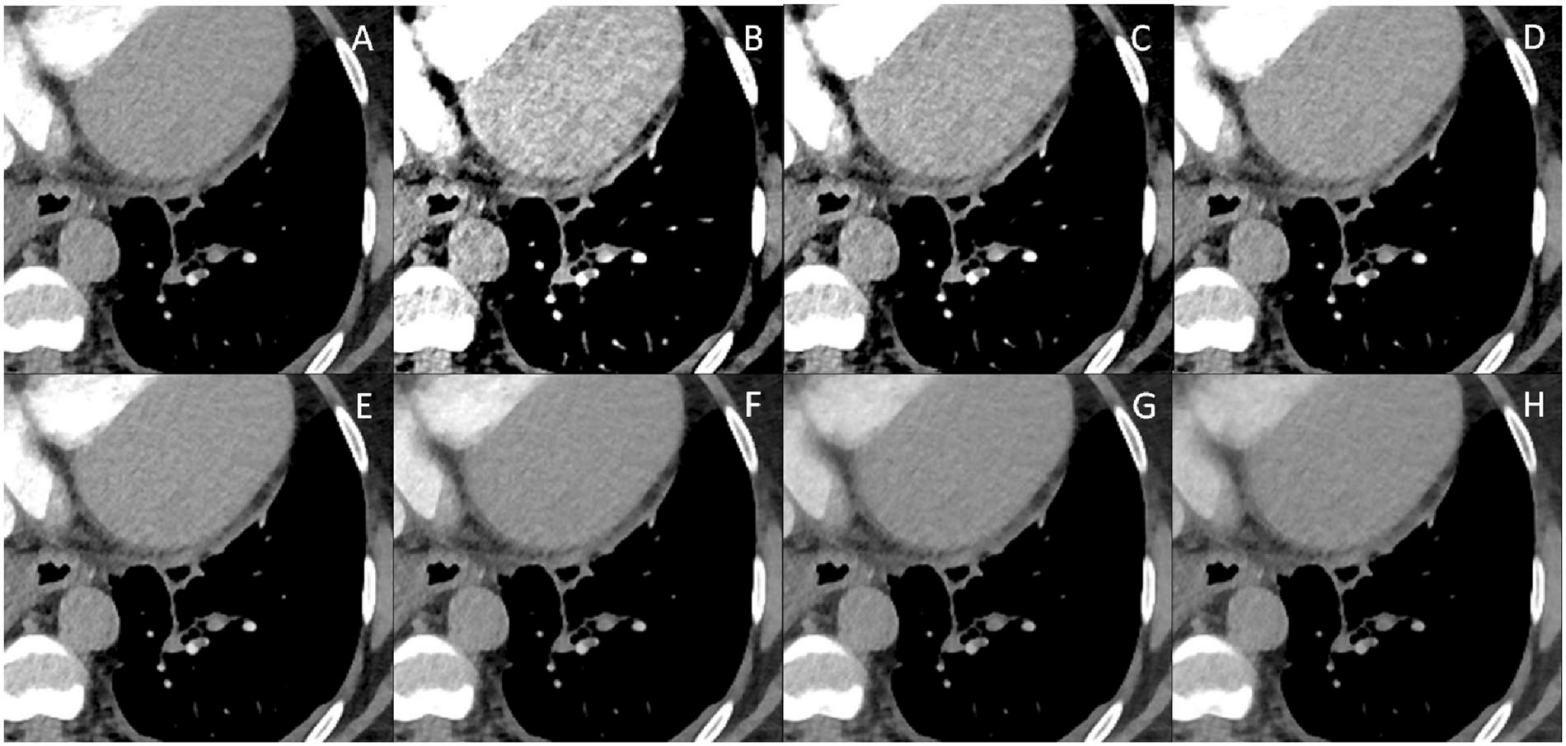
62-year-old woman with suspected pulmonary embolism. Axial slices of peripheral pulmonary arteries of a low contrast media dual-energy CTPA: (**A**) mixed 0.8-weighted virtual polyenergetic spectral (VPS) image, and virtual monoenergetic spectral (VMS) images at a level of 40 keV (**B**), 50 keV (**C**), 60 keV (**D**), 70 keV (**E**), 80 keV (**F**), 90 keV (**G**) and 100 keV (**H**). The VMS image at 50 keV displays superior subjective image quality when compared to VPS image.

No differences regarding background noise could be observed between the three protocols (standard CTPA 12.1 ± 2.1 HU vs. standard DE CTPA 10.4 ± 1.8 HU vs. low CM DE CTPA 14.3 ± 2.3 HU; for all p > 0.05). Vessel attenuation (enhancement) and image noise increased with decreasing energy levels for the 40–100 keV VMS datasets (compare Figs 2–4).

The 70 keV VMS dataset for the main pulmonary arteries showed the highest SNR (standard DE CTPA: 23.0 ± 9.1 vs. low CM DE CTPA 17.9 ± 2.8; p < 0.0001). The 50 keV dataset showed the highest CNR for both DE CTPA protocols, with significantly higher values for the standard DE CTPA protocol (p < 0.0001). When compared to the standard CTPA protocol datasets, CNR was significantly higher for both DE CTPA protocol VMS datasets at 50 keV (both p < 0.0073).

For the peripheral pulmonary arteries, the 50 keV VMS dataset of the low CM DE CTPA protocol showed the highest SNR and the 70 keV dataset of the standard DE CTPA protocol (standard DE CTPA: 15.6 ± 6.2 and low CM DE CTPA 14.0 ± 6.1). The 40 keV dataset revealed the highest CNR for the low DE CTPA protocol (p > 0.05) and for the standard DE CTPA protocol both 40 and 50 keV revealed the highest CNR. Similarly, when comparing the standard protocol to the 40 and 50 keV VMS datasets, there was a significant increase in CNR for both DE CTPA protocols (both p < 0.0064).

Table 2 summarizes the objective image quality assessment for the standard CTPA protocol, the standard DE CTPA and the low CM DE CTPA protocol datasets.

**Table 2:** Objective and subjective image quality between DE CTPA VMS and the standard CTPA datasets.

	Low CM DECT		Standard CM DECT		Standard CTPA	p-value			
	40 *keV[A]*	50 *keV[B]*	40 *keV[C]*	50 *keV[D]*	*[E]*				
*Main pulmonary arteries*						*A vs C*	*B vs D*	*B vs E*	*D vs E*
Attenuation	649.2 ± 59.7	437.4 ± 39.5	850.2 ± 242.5	575.3 ± 161.3	375.7 ± 120.3	<0.0001	<0.0001	0.0022	<0.0001
Signal-to-noise-ratio	16.1 ± 2.8	16.8 ± 2.8	21.5 ± 9.1	22.1 ± 9.3	14.0 ± 3.7	<0.0001	<0.0001	0.0002	<0.0001
Contrast-to-noise-ratio	14.4 ± 2.4	14.5 ± 2.4	20.7 ± 9.1	20.8 ± 9.1	12.7 ± 3.5	<0.0001	<0.0001	0.0073	<0.0001
Subjective image quality	4[4–5]	5[4–5]	4[4–5]	5[4–5]	5[4–5]	0.8762	0.7335	0.6581	0.6632
Subjective image noise	4[3–4]	4[3–5]	4[3–4]	4[4–5]	4[3–5]	0.8571	0.7684	0.8039	0.1344
*Peripheral pulmonary arteries*						*A vs C*	*B vs D*	*A vs E*	*C vs E*
Attenuation	545.4 ± 74.2	358.5 ± 48.0	857.4 ± 246.0	580.1 ± 163.5	358.8 ± 110.4	<0.0001	<0.0001	<0.0001	<0.0001
Signal-to-noise-ratio	13.8 ± 6.2	14.0 ± 5.9	14.6 ± 6.3	15.1 ± 6.4	9.7 ± 4.5	0.4045	0.3017	<0.0001	<0.0001
Contrast-to-noise-ratio	12.1 ± 5.5	11.7 ± 5.1	14.1 ± 6.3	14.1 ± 6.3	8.8 ± 4.2	0.1466	0.0538	0.0064	<0.0001
Subjective image quality	5[4–5]	5[4–5]	5[4–5]	5[4–5]	5[4–5]	0.7976	0.8112	0.3475	0.7214
Subjective image noise	4[3–4]	4[3–5]	4[3–4]	4[3–5]	4[3–4]	0.9117	0.8549	0.1224	0.1463

Note VMS = virtual monoenergetic spectral; CTPA = CT pulmonary angiography; keV = kiloelectron Volt; values in brackets represent the 25–75% interquartile.

### Comparison of subjective image quality

There was a substantial agreement between both radiologists for image quality rating (κ = 0.71). No significant difference could be detected in the median image quality or the median image noise for both the 40 keV and 50 keV VMS dataset (all p > 0.05) when comparing the subjective image quality between both DE CTPA protocol VMS datasets for the main pulmonary arteries and the peripheral pulmonary arteries. Likewise, there was no significant difference in the median image quality or median image noise for both DE CTPA 40 keV and the 50 keV protocols compared to the standard CTPA protocol (all p > 0.05).

### Diagnostic accuracy

No false positive or false negative findings were revealed considering the 40 keV and 50 keV VMS datasets of the standard DE CTPA, resulting in a sensitivity, specificity and accuracy of 100%. Analogically, considering the 40 keV and 50 keV VMS datasets of the low CM DE CTPA, no false positive or false negative findings were observed, resulting in a sensitivity, specificity and accuracy of 100%.

### Patient serum creatinine levels before and after the CTPA protocols

On the 24 hours and 72 hours follow-up, none of the 150 patients suffered from CIN, as serum creatinine levels were similar for the low CM DE CTPA group (mean difference after 72 h −0.02 mg/dl; p > 0.05). Serum creatinine levels slightly decreased/increased after 72 h for both standard CTPA groups compared to the baseline evaluation (DE standard CTPA: mean difference after 72 h −0.02 mg/dl and standard CTPA mean difference after 72 h + 0.06 mg/dl; both p > 0.05). In a follow-up serum creatinine evaluation after 3 months, serum creatinine levels again showed no differences to the baseline evaluation for the standard CM dose CTPA protocols (all p > 0.05) and significantly lower values on 3 month follow-up for the low CM DE CTPA group, mainly because of the normalization of serum creatinine levels of those patients with acute kidney failure due to other reasons. However, due to the small sample size, it has to be noted that the statistical power is not sufficient with π = 0.72.

## Discussion

The results of this study demonstrate that DE CTPA studies are feasible using only 5.4 g of iodine with a non-inferior image quality when compared to both a standard CTPA and a DE CTPA protocol with a standard CM dose. In addition, no significant increase in serum creatinine levels between baseline and 24–72 hours follow-up within the low CM DE CTPA group was observed, whereas both other groups showed slightly increased serum creatinine levels. However, it is important to point out that none of our patients required temporary hemodialysis during three months of follow-up, which supports the results of the current literature stating that AKI is most likely independent from intravenous CM administration amongst all risk groups^3–6^.

Vessel opacification is mandatory for diagnostic image quality, allowing for diagnostic confidence. In general, an attenuation of 180 HU and higher is regarded as diagnostically sufficient for pulmonary arteries^23,24^. By using a DECT protocol, it is possible to extrapolate images close to a monochromatic x-ray beam of various energy levels. This allows the selection of ideal photon energy levels with regard to the evaluation of vascular compartments^25^. Similarly, recent studies have shown improved attenuation and visualization of vascular structures at low kVp levels in a chest CTA^26–29^.

Depending on the DECT technique used, recent studies advocated 60–70 keV and 50–70 keV for an optimal CNR of the pulmonary arteries for dual source and fast kVp switching DE techniques, respectively^10,11,13,30^. So far however, studies performed on a DSCT have used a first generation monoenergetic algorithm, which causes a significant increase in image noise for low VMS datasets. This limits diagnostic image quality and confidence, especially in the 40–60 keV VMS datasets^10,11,30^. In contrast to the above mentioned studies, our study demonstrates the best CNR at 50 keV for the main pulmonary arteries and at 40 keV for the peripheral pulmonary arteries, regardless of the contrast media applied or the generation of DSCT. This is mainly linked to the second generation monoenergetic algorithm with a re-developed noise reduction filter utilizing a frequency split technique, which reduces image noise for the lower VMS datasets^19^.

VMS datasets at an optimal keV level improve vessel attenuation of pulmonary arteries, allowing for lower volumes of contrast media. Administration of intravenous CM in patients with renal risk factors remains subject to debate in everyday clinical routine, although several recent studies reported that CIN might be independent from the administration of intravenous CM amongst different risk groups^3–6^. However, CIN has accounted for up to 12% of all hospital-acquired acute renal failures in the past and has been associated with a considerable mortality and morbidity rate^31^. So far, data and knowledge for patients undergoing several CT examinations within several days, as well as a potential dose dependency between intravenous CM and AKI remains insufficient. Therefore, lowering the amount of contrast media is recommended by current guidelines^2^ and one of the most effective methods of reducing potential contrast media induced cytotoxic effects. Using the introduced novel protocol in this study might be a valid option for high-risk patients, for whom clinicians would order a contrast-enhanced CT more conservatively^31^.

Several studies have evaluated low contrast media CTPA protocols with iodine loads ranging between 9–30 g^9,11,13,30,32^. However, most of these studies have used a test bolus in order to achieve a low contrast media protocol. This is an unnecessary contrast media application without diagnostic information, thus exposing patients with renal risk factors to additional contrast dose. Furthermore, the above mentioned studies are not entirely explicit whether the iodine amounts included the iodine load of the test bolus, which ranges between 3.7 g and 7 g. In contrast, the low CM DE CTPA protocol used in this study, utilizing a bolus tracking protocol, has only required a total iodine load of 5.4 g to achieve sufficient vessel opacification. This amount is substantially lower compared to previous investigations. None of our patients suffered from a CIN 24 hours and 72 hours after CT examination, although 60% of our patient cohort had known acute or chronic renal insufficiency.

The effective dose of 3.4 mSv for the low CM DECT CTPA protocol in this study was significantly higher than the 2.5 mSv of the standard CTPA. However, patients with acute or chronic renal failure may benefit from this protocol, as long as CIN due to intravenously administered CM is not completely ruled out. Therefore, the benefits of a reduced CM amount have to be outweighed against the risk for radiation-induced cancer. The attributed 5-year survival rate of patients with arteriovenous fistulas/ arteriovenous grafts due to end stage renal impairment is 36%^33^. The relative cancer risk lies between 1.001 and 1.04 for radiation doses of <100 mSv with an expected onset 15 to 20 years after exposure^34^.

The effective dose of 3.4 mSv for the low CM DE CTPA protocol was lower than that reported in previous DECT CTPA protocol investigations, which ranged between 4.6–7.0 mSv^11,13,30^, and the standard DE CTPA protocol used in this study, which had an effective dose of 3.9 mSv. This is mainly linked to using a 3^rd^ generation DSCT system. This system combines a more efficient detector design with automated tube modulation selection, as well as a 3^rd^ generation IR technique to reduce the applied radiation dose. In the near future, when photon-counting detector CT systems become clinically available, both a reduced radiation exposure and a reduced amount of CM may become feasible by k-edge subtraction imaging while maintaining high image quality.

As all patients of the DE CTPA protocol had acute or chronic renal failure, the trade-off between radiation and contrast media amount shifts toward the lowest possible total iodine dose, rather than the lowest possible radiation dose. Nonetheless, each DECT examination should comply with the as low as reasonably achievable principle with reasonable radiation dose.

Several study limitations have to be noted. First of all, our primary focus in this study was to evaluate objective and subjective image criteria of a low iodine CM CTPA protocol. Therefore, a control scan with a standard CM amount in order to determine false positive or false negative findings was not performed due to ethical considerations regarding total amounts of CM and additional radiation dose. We counterbalanced this with a strict standard of reference. Furthermore, the sufficient vessel attenuation of 180 HU and above was achieved, as well as increased CNR values of the 40 and 50 keV VMS datasets compared to the standard CTPA protocol. This allowed a decision-making with sufficient diagnostic confidence for confirming or ruling out a PE. Secondly, we did not evaluate or document any specific cardiac output parameters, which may have a considerable effect on vascular enhancement in CTPA^35^. Thirdly, there is a selection bias in our study, as we primarily included patients with acute or chronic renal failure in the low CM DE CTPA group, as there was only a low number of patients with renal risk factors in the other two groups. This might confound our exploratory assessment of CIN. Furthermore, it was reported that patients with acute and chronic renal failure have a higher risk of venous thromboembolism^36^, which may be one reason for the relative high incidence of PE in this study group. Finally, the CTPA scans were performed on different CT scanners with different scan settings, which might influence image quality. However, the purpose of this study was to demonstrate the implementation of a low CM protocol, which results in similar CNR as a standard CTPA with a standard CM protocol.

In conclusion, we have demonstrated that a DE CTPA protocol performs equally to a standard CTPA protocol and can be used to save contrast media when using VMS reconstructions of low energy datasets without a reduction in diagnostic accuracy, vessel opacification, CNR or perceived image quality. VMS datasets of 40–50 keV are ideal when reducing the total iodine amount down to 5.4 g (reduction of 83%). However, the acquisition using a DE CTPA protocol comes at the disadvantage of an increased radiation exposure of 26% compared to a reduced tube voltage standard CTPA protocol of 100 kVp. Therefore, the benefits of a reduced CM amount have to be outweighed against the risk for radiation-induced cancer.
